# Re-evaluating the evidence for facilitation of stickleback speciation by admixture in the Lake Constance basin

**DOI:** 10.1038/s41467-021-23092-1

**Published:** 2021-05-14

**Authors:** Daniel Berner

**Affiliations:** Department of Environmental Sciences, Zoology, University of Basel, Basel, Switzerland

**Keywords:** Evolutionary genetics, Population genetics, Adaptive radiation

**Arising from** Marques et al. *Nature Communications* https://doi.org/10.1038/s41467-019-12182-w (2019)

Where genetic variation promoting speciation originates is a crucial question in evolutionary genomics. In a recent article, Marques et al.^1^ seek to address this question in lake and stream threespine stickleback fish from the Lake Constance (hereafter referred to as LC) basin in Central Europe. Based on population genetic methods, they conclude that incipient speciation between lake and stream stickleback was facilitated by the recent mixing of genetic variation from old lineages evolved in isolation (i.e., admixture following secondary contact). In this comment, I discuss conceptual and methodological problems and unrecognized conflicts with existing evidence that cast doubt on Marques et al.’s conclusion.

## The origin of stickleback in the LC basin

Marques et al. argue that threespine stickleback populations in the LC basin result from a contact between two deeply separated lineages from Northeastern and Western Europe, and that anthropogenic introduction played an important role in the colonization of the LC basin. I here revisit these views based on a comprehensive nuclear phylogeny for Central European stickleback.

**Fig. 1:**
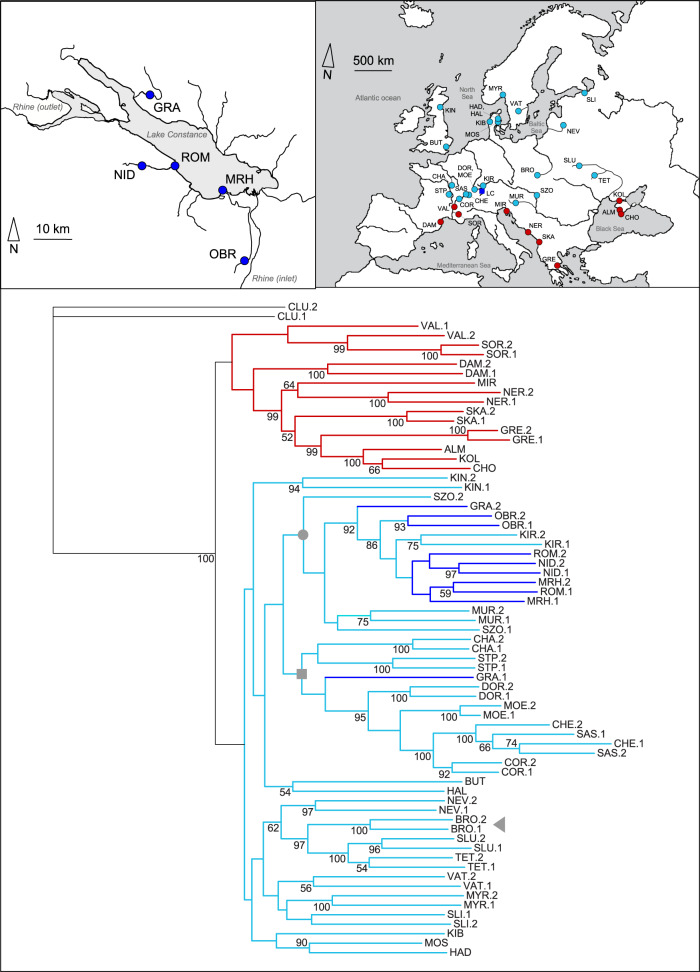
Phylogeny of European threespine stickleback populations. The phylogram (maximum likelihood tree) is based on DNA sequence data from 69 total stickleback individuals from the 39 freshwater populations indicated in the maps (1–2 individuals per population). The left map represents a close-up of the Lake Constance (LC) region, located by a dark blue square in the right map, showing the precise situation of the two lake (ROM and MRH) and three stream (GRA, NID, and OBR) sample sites. The color coding separates the populations belonging to the circum-Mediterranean and Black Sea lineage (red) from those belonging to the Central, Northern, and Eastern European lineage (blue; populations from the LC basin are labeled in dark blue). The values next to nodes give the strength of monophyly of the corresponding branches based on bootstrapping (500 iterations; shown only for values ≥50%). Note the strong bootstrap support for the reciprocal monophyly of the two major (red, blue) stickleback lineages in Europe. By contrast, the basal nodes within the blue lineage lack bootstrap support, thus challenging Marques et al.’s assumption of an old, genetically distinct Western European lineage (the basal node of this lineage is marked by a gray square). The branch marked by the gray dot contains exclusively populations from the LC basin and the Danube river, highlighting their close genetic relatedness. The gray triangle indicates the BRO population chosen by Marques et al. as representative of their Northeastern European lineage. Two individuals derived from Pacific ancestors (CLU) served as the outgroup.

This phylogeny (Fig. 1) confers two major insights: first, European stickleback populations separate deeply into a Mediterranean and Black Sea lineage on the one hand, and a Central, Eastern, and Northern European lineage on the other hand (for evidence based on ordination see Supplementary Fig. 1). This dichotomy is consistent with a recent phylogeographic investigation^2^ establishing that the circum-Mediterranean and Black Sea lineage reflects an ancient southern refugial ancestor, whereas the more northern populations derive from a large-scale postglacial surge in southwestward direction via an ancient Baltic Sea. However, the phylogeny does not support Marques et al.’s claim of the existence of an ancient, genetically distinct Western European stickleback lineage “evolved in isolation for several thousand generations”^1^: statistical support for the monophyly of the authors’ western lineage (indicated by a gray square in Fig. 1) is poor, and the basal branch of this western lineage is not deeper than branches representing populations from other drainages in Northern or Eastern Europe.

The second insight from the phylogeny is that stickleback from the LC basin prove closely related to populations from the Danube drainage (Fig. 1 and Supplementary Fig. 1). The broad-scale colonization history of Central Europe^2^ in mind, this close genetic relatedness supports the possibility that stickleback in the LC basin may originate from the natural westward colonization by Northeastern European (but not Black Sea^2,3^) stickleback via the Danube drainage. The LC basin nowadays drains into the Atlantic via the river Rhine, so its colonization via the Danube drainage may appear counter-intuitive. However, during the retreat of the Pleistocene ice cover, the present-day LC basin drained in an eastward direction via the Danube^4^. Even today, the Danube and the LC drainage remain connected through a sinkhole and a 12 km underground stream system^5^ inhabited by fish^6^. Evidence of a natural, postglacial colonization of the LC basin also emerges from the authors’ own demographic analysis: their deepest splitting time estimate between populations from the LC basin is 2800 generations (a generation is 1–2 years^3^) before present, a value in line with an earlier estimate for stream populations in the LC basin (~2300 generations, Supplementary Fig. 2 in ref. ^7^; note, however, that the studies differ in the mutation rates assumed). While the authors recognize that these estimates conflict with a very recent anthropogenic origin, they consider shortcomings in their demographic modeling, but not the possibility that at least the stream ecotype may have colonized the LC basin naturally thousands of generations ago.

Overall, Marques et al.’s assumption of a Western European stickleback lineage with a long history of evolution in isolation, and the view that recent introductions were important to the establishment of stickleback in the LC region, do not appear well supported by phylogenetic evidence. Although my analyses include new sequence data not available to the authors’ original investigation, analogous analyses considering only data contemporaneous with their work also raise the above concerns (Supplementary Fig. 2). In this light, it is hard to follow why the authors did not aim for more complete population coverage (especially including Danubian stickleback) for their genetic inference.

## Overconfidence in population genetic methods

Another problem in Marques et al. is that strong conclusions about evolutionary history are derived from population genetic analyses without carefully acknowledging potential violations of the underlying assumptions, and ambiguity in their interpretation. A major issue is that their main methodological tools (demographic modeling, *D* statistic) assume selectively neutral evolution. However, natural selection imposed by novel local ecological conditions profoundly re-structures genetic variation all across the stickleback genome^8^. That such selection has the potential to bias demographic inference has been suggested in stickleback from the LC basin^7^. For fairness, it must be highlighted that Marques et al. attempt to reduce potential bias due to selection in their demographic analyses by excluding markers located in chromosome regions exhibiting a particularly low recombination rate. While this data manipulation may increase analytical robustness in older organismal systems in which genomic variation is shaped by background selection (a mutation-driven process), it may be less effective in a young, postglacial system like stickleback strongly influenced by the rapid directional selection of standing genetic variation. This skepticism is confirmed directly by a recent genomic analysis of lake-stream stickleback from the LC basin based on whole-genome marker resolution, revealing that signatures of divergent selection are neither less common nor less extensive physically in the chromosome peripheries exhibiting high recombination rates^9^ (Fig. 2 and Supplementary Fig. 1 in ref. ^10^). Likewise, it is easy to imagine that selection can bias *D* statistics: Marques et al. argue that Northeastern European stickleback are phenotypically closer to ancestral marine stickleback than are Central European populations. However, stickleback within LC are selected for a pelagic lifestyle resembling that of marine fish^3,7^, hence greater allele sharing between the LC population and Northeastern European populations is an expected outcome of local adaptation potentially confounding the inference of admixture.

Also, Marques et al. claim to evaluate several possible demographic scenarios for the colonization and subsequent divergence of stickleback in the LC basin, and in particular to refute an “ecological vicariance” scenario (Fig. 3 in ref. ^7^). An inherent element of ecological vicariance, however, is population differentiation caused by strong divergent selection, for which there is clear experimental evidence in lake and stream fish from the LC basin^10,11^. Marques et al.’s demographic analysis, assuming the absence of selection, must therefore fail to offer an adequate comparison of relevant evolutionary scenarios. Another source of concern is that their demographic analysis assumes that all populations had constant sizes during evolution. Fluctuations in population sizes, however, can bias demographic model selection toward secondary contact scenarios^12^. These methodological caveats in mind, Marques et al.’s inference of an admixture history must be regarded as speculative and no more plausible than alternative scenarios.

## Facilitation of speciation?

A final issue is that even if we assume that admixture between distinct lineages has occurred in the LC basin, the evidence presented by Marques et al. is insufficient for demonstrating that this has promoted divergence. A challenge is that within the LC basin, the lake population has adapted relatively recently to the pelagic ecological niche and represents the most derived ecotype in that region, while stream-adapted populations are ancestral^7^. Admixture would thus be expected to promote speciation only if it introduced genetic variation for pelagic adaptation not previously present in the basin already. However, such variation is unlikely to come from the authors’ western lineage, which includes only stream-adapted populations. Admitting the possibility that a stream-adapted population in one watershed may still hold genetic variation useful to pelagic adaptation in another watershed, this ecological ambiguity calls for a direct demonstration of admixture as a driver of speciation. Specifically, one would need to (i) identify the specific haplotypes (DNA sequence stretches) holding alleles involved in divergent adaptation, and (ii) demonstrate that such haplotypes were initially missing in one or the other original population.

However, none of the authors’ genetic population samples from outside the LC basin include more than seven individuals, thus precluding robust estimates of haplotype frequencies needed for inferring variational constraints broken by admixture. More fundamentally, the sparse marker resolution of Marques et al. is insufficient for haplotype-level inference in the first place^13^. However, for a few genome regions under divergent lake-stream selection, genotype data at the level of phased haplotypes have been generated previously by targeted sequencing in stickleback from both the LC basin and the authors’ western lineage. These regions include the EDA locus underlying variation in body plating (Fig. 5b in ref. ^14^), and three large inversions (Fig. 7c in ref. ^7^). These data demonstrate that the genetic variants underlying lake-stream divergence within the LC basin are not only ubiquitous across Europe, but shared among populations on a worldwide scale. The strongest sequence-based evidence currently available thus indicates that lake and stream stickleback within the LC basin have diversified just as stickleback populations do everywhere: by sorting abundant standing genetic variation preexisting in their ancestors. Conclusively evaluating a potential contribution of admixture to adaptive diversification and speciation in this system would require a methodological stringency beyond the standard of Marques et al.’s work.

To conclude, in view of the problems highlighted in this note, Marques et al.’s claim to “have demonstrated that secondary contact between divergent lineages and the re-assortment of introgressed alleles […] underlie recent ecological speciation” seems overconfident and lacking convincing empirical evidence.

## Methods

The phylogenetic analysis involves stickleback samples from 39 localities in and around central Europe, most of which are represented by two individuals (69 individuals in total; Supplementary Table 1). Because of strong adaptive genetic divergence between marine and freshwater stickleback populations, all 38 European localities concern exclusively freshwater habitat; only a single locality (CLU; Cluxewe Estuary, Vancouver Island, Canada) included as an outgroup represents saltwater habitat (anadromous marine stickleback). All populations are at least potentially natural, except for the populations CHE and SAS, which originate from human introduction to the Lake Geneva basin ~1900^15,16^. The data underlying this analysis are *Sbf*I or *Pst*I enzyme restriction site-associated DNA (RAD) sequences generated specifically for this study (four localities), or retrieved from published investigations^1,2,7,17–20^. In the latter case, the two individuals with the highest read depth were given priority when more than two individuals were available from a given locality. The full data set is described in detail in Supplementary Table 1.

Because some of these sequence data were not available to the investigation published in Marques et al.^1^, I examined whether a phylogenetic analysis based exclusively on data contemporaneous to that study and known to the authors produced qualitatively similar conclusions. Specifically, I here considered the data subset from Marques et al.^1^ and Fang et al.^2^ only, noting that the latter study was used by Marques et al. as a source of sequence data for a few selected populations (ALM, CHO, and KOL). This reduced analysis included 53 total individuals from 29 localities (including the same outgroup as the full analysis).

Using the R package *ShortRead*^21^, all raw fastq files were initially filtered for reads starting with the exact *Sbf*I restriction residual (TGCAGG; *Sbf*I restriction sites are covered by the *Pst*I enzyme too), and the reads were trimmed to 70 base pairs (bp). The fastq data thus obtained were aligned to the threespine stickleback reference genome assembly^22^ with Novoalign v3.00 (http://www.novocraft.com/products/novoalign), using the alignment parameters from ref. ^23^ (key settings: -t180 -g40 -x15). To obtain single-nucleotide polymorphisms (SNPs) for phylogenetic analysis, the alignments were then converted to BAM format and processed by using the R packages *Rsamtools*^24^ and *stringr*^25^. At each RAD locus in each individual, haploid genotyping was performed by retrieving the leading haplotype, defined as the single sequence exhibiting the highest read count among all unique sequences present at the RAD locus. RAD loci at which this leading haplotype did not occur in at least two copies, or exhibiting an excessive read depth beyond 4.5 times the expected read depth across all genome-wide RAD loci (estimated by the total number of reads divided by the total number of RAD loci), were excluded from the analysis. This haploid genotyping approach was chosen because it avoids potential bias in the identification of heterozygous positions and is therefore highly reliable. RAD loci successfully genotyped in every single individual were then used for SNP detection (hence, the final SNP data set contained no missing data). I accepted SNPs along a RAD locus only if they were at least 8 bp away from the previous polymorphic position, thus avoiding pseudo-SNPs arising from indels. For each individual, the nucleotides present at all SNPs were concatenated to a single string, and these strings combined across all individuals in a single fasta file. For the analysis using the complete data set (39 localities), the fasta file contained genotype information from 7121 SNPs from 4429 genome-wide RAD loci shared among all individuals, while for the reduced analysis (29 localities), 7616 SNPs from 4961 RAD loci were available.

As a robustness check, the above SNP detection and genotyping protocol was repeated for both the complete and reduced data set by using more stringent quality filters: for an individual, the leading haplotype at a RAD locus was accepted only if present in at least five (as opposed to two) copies, and the minimum spacing threshold for SNPs located on the same RAD locus was increased from 8 to 12 bp. This latter approach, yielding 797 SNPs from 563 RAD loci for the complete and 1161 SNPs from 824 loci for the reduced analysis, produced very similar phylogenetic tree topologies leading to the same conclusions (details not presented).

Phylogenetic analysis based on the above fasta files was carried out using the R packages *ape*^26^ and *phangorn*^27^. I first determined the most appropriate substitution model (GTR + G + I), estimated the maximum likelihood tree, and visualized this tree as phylogram. The phylogenies were complemented by visualization of genetic similarity among individuals using ordination. I here analyzed genetic distance matrices derived from the fasta files using principal coordinates analysis (PCoA). These supplementary analyses, performed for both the complete and reduced data sets, considered the European individuals only; the two CLU individuals from Canada were excluded. Individuals were plotted along the first two PCoA axes. All analyses were performed in R^28^.

### Reporting summary

Further information on research design is available in the Nature Research Reporting Summary linked to this article.

## Supplementary information

Supplementary Information

Description of Additional Supplementary Files

Supplementary Code 1

Reporting Summary

## Data Availability

All raw Illumina sequence data newly generated for this study are available from the NCBI Sequence Read Archive under BioProject PRJNA566094 (accession numbers: SRX6864080, SRX6864081, SRX6864092, SRX6864103, SRX6864114, SRX6864125, SRX6864129, SRX6864130). Accession numbers of the reused sequence data are listed in Supplementary Table 1.

## Code availability

A collection of all codes used for data analysis is provided as Supplementary Code 1.

## References

[1] Marques DA, Lucek K, Sousa VC, Excoffier L, Seehausen O (2019). Admixture between old lineages facilitated contemporary ecological speciation in Lake Constance stickleback. Nat. Commun..

[2] Fang B, Merilä J, Ribeiro F, Alexandre CM, Momigliano P (2018). Worldwide phylogeny of three-spined sticklebacks. Mol. Phylogenet. Evol..

[3] Moser D, Roesti M, Berner D (2012). Repeated lake-stream divergence in stickleback life history within a Central European lake basin. PLoS ONE.

[4] Keller O, Krayss E (2000). Die Hydrographie des Bodenseeraums in Vergangenheit und Gegenwart. Ber. St. Gall. Naturwiss. Ges..

[5] Hötzl H (1996). Origin of the Danube-Aach system. Environ. Geol..

[6] Behrmann-Godel J, Nolte AW, Kreiselmaier J, Berka R, Freyhof J (2017). The first European cave fish. Curr. Biol..

[7] Roesti M, Kueng B, Moser D, Berner D (2015). The genomics of ecological vicariance in threespine stickleback fish. Nat. Commun..

[8] Bassham S, Catchen J, Lescak E, von Hippel FA, Cresko WA (2018). Repeated selection of alternatively adapted haplotypes creates sweeping genomic remodeling in stickleback. Genetics.

[9] Roesti M, Moser D, Berner D (2013). Recombination in the threespine stickleback genome - patterns and consequences. Mol. Ecol..

[10] Laurentino TG (2020). Genomic release-recapture experiment in the wild reveals within-generation polygenic selection in stickleback fish. Nat. Commun..

[11] Moser D, Frey A, Berner D (2016). Fitness differences between parapatric lake and stream stickleback revealed by a field transplant. J. Evol. Biol..

[12] Momigliano, P., Florin, A.-B. & Merilä, J. Biases in demographic modelling affect our understanding of recent divergence. *Mol. Biol. Evol.* (in the press). 10.1093/molbev/msab047 PMC8233503 33624816

[13] Lowry DB (2017). Breaking RAD: an evaluation of the utility of restriction site-associated DNA sequencing for genome scans of adaptation. Mol. Ecol. Res..

[14] Berner D, Roesti M, Hendry AP, Salzburger W (2010). Constraints on speciation suggested by comparing lake-stream stickleback divergence across two continents. Mol. Ecol..

[15] Fatio, V. *Faune des Vertébrés de la Suisse - Histoire naturelle des Poissons* (H. Georg, 1882).

[16] Bertin, L. *Recherches bionomiques, biométriques et systématiques sur les épinoches (Gastérostéidés*) (Blondel La Rougery, 1925).

[17] Roesti M, Salzburger W, Berner D (2012). Uninformative polymorphisms bias genome scans for signatures of selection. BMC Evol. Biol..

[18] Roesti M, Gavrilets S, Hendry AP, Salzburger W, Berner D (2014). The genomic signature of parallel adaptation from shared genetic variation. Mol. Ecol..

[19] Ferchaud A-L, Hansen MM (2016). The impact of selection, gene flow and demographic history on heterogeneous genomic divergence: threespine sticklebacks in divergent environments. Mol. Ecol..

[20] Marques DA (2016). Genomics of rapid incipient speciation in sympatric threespine stickleback. PLoS Genet..

[21] Morgan M (2009). *ShortRead*: a Bioconductor package for input, quality assessment and exploration of high-throughput sequence data. Bioinformatics.

[22] Glazer AM, Killingbeck EE, Mitros T, Rokhsar DS, Miller CT (2015). Genome assembly improvement and mapping convergently evolved skeletal traits in sticklebacks with genotyping-by-sequencing. G3.

[23] Roesti M, Hendry AP, Salzburger W, Berner D (2012). Genome divergence during evolutionary diversification as revealed in replicate lake-stream stickleback population pairs. Mol. Ecol..

[24] Morgan, M., Pages, H., Obenchain, V. & Hayden, N. *Rsamtools*: binary alignment (BAM), FASTA, variant call (BCF), and tabix file import. http://bioconductor.org/packages/release/bioc/html/Rsamtools.html (2017).

[25] Wickham, H. *stringr*: simple, consistent wrappers for common string operations. https://CRAN.R-project.org/package=stringr (2019).

[26] Paradis E, Schliep K (2018). *ape* 5.0: an environment for modern phylogenetics and evolutionary analyses in R. Bioinformatics.

[27] Schliep K (2011). *phangorn*: phylogenetic analysis in R. Bioinformatics.

[28] R Core Team. *R: A Language and Environment for Statistical Computing* (R Foundation for Statistical Computing, 2020).

