# Supplementary material for: Re-evaluating the evidence for facilitation of stickleback speciation by admixture in the Lake Constance basin
Source: Nat Commun. 2021 May 14;12:2806. doi: 10.1038/s41467-021-23092-1 (PMC8121923; doi:10.1038/s41467-021-23092-1)
Supplement: Supplementary file 1 — Supplementary Information [file 41467_2021_23092_MOESM1_ESM.pdf]

Supplementary Information to

Re-evaluating the evidence for facilitation of stickleback speciation by admixture in the Lake Constance basin

Daniel Berner

Contents:

- **Supplementary Table 1**
- **Supplementary Fig. 1**
- **Supplementary Fig. 2**
- **Supplementary References**

Supplementary Table 1. Description of the RAD sequence data underlying the population genetic analyses. Accession codes refer to the NCBI Sequence Read Archive.

Individual	Locality	Country	Restriction enzyme	Accession	Reference
ALM	Alma	Ukraine	Pst1	SRX3998066	1
BRO.1	Broszkowice	Poland	Sbf1	SRX6084984	2
BRO.2	Broszkowice	Poland	Sbf1	SRX6084934	2
BUT	Butler pond	UK	Pst1	SRX3997979	1
CHA.1	Butler pond	France	Pst1	SRX3997970	1
CHA.2	Chamoux	France	Pst1	SRX3997983	1
CHE.1	Chessel	Switzerland	Sbf1	SRX1038796	3 (locality described in ref. 4)
CHE.2	Chessel	Switzerland	Sbf1	SRX1038804	3 (locality described in ref. 4)
CHO	Chornaya	Ukraine	Pst1	SRX3998069	1
CLU.1	Cluxewe Estuary, Vancouver Island	Canada	Sbf1	SRX456700	5
CLU.2	Cluxewe Estuary, Vancouver Island	Canada	Sbf1	SRX456701	5
COR.1	Cormoz	France	Sbf1	SRX6084965	2
COR.2	Cormoz	France	Sbf1	SRX6084963	2
DAM.1	Treilles	France	Pst1	SRX3998041	1
DAM.2	Treilles	France	Pst1	SRX3998036	1
DOR.1	Basel	Switzerland	Sbf1	SRX6864080	this study (locality described in ref. 6)
DOR.2	Basel	Switzerland	Sbf1	SRX6864081	this study (locality described in ref. 6)
GRA.1	Grasbeuren	Germany	Sbf1	SRX6864092	this study (locality described in ref. 6)
GRA.2	Grasbeuren	Germany	Sbf1	SRX6864103	this study (locality described in ref. 6)
GRE.1	Spercheios	Greece	Pst1	SRX3998070	1
GRE.2	Spercheios	Greece	Pst1	SRX3998071	1
HAD	Hadsten	Denmark	Sbf1	SAMN03076274 (Had3)	7
HAL	Hald	Denmark	Sbf1	SAMN03076274 (Hal3)	7
KIB	Kibaek Molledam	Denmark	Sbf1	SAMN03076274 (Kib7)	7
KIN.1	Kinness Burn	UK	Pst1	SRX3998013	1
KIN.2	Kinness Burn	UK	Pst1	SRX3998012	1
KIR.1	Kirchbierlingen	Germany	Sbf1	SRX6864114	this study (locality described in ref. 6)
KIR.2	Kirchbierlingen	Germany	Sbf1	SRX6864125	this study (locality described in ref. 6)
KOL	Kolanraes	Ukraine	Pst1	SRX3998068	1
MIR	Mirna	Croatia	Pst1	SRX3998033	1
MOE.1	Moehlin	Switzerland	Sbf1	SRX6084954	2
MOE.2	Moehlin	Switzerland	Sbf1	SRX6084993	2
MOS	Mosso	Denmark	Sbf1	SAMN03076274 (Mos4)	7
MRH	Marina Rheinhof	Switzerland	Sbf1	SRS1271407	8
MRH	Marina Rheinhof	Switzerland	Sbf1	SRS1271408	8
MUR.1	Mura	Slowenia	Pst1	SRX3997988	1
MUR.2	Mura	Slowenia	Pst1	SRX3997989	1
MYR	Myrdalsvatnet	Norway	Pst1	SRX3998020	1
MYR	Myrdalsvatnet	Norway	Pst1	SRX3997992	1
NER.1	Neretva	Croatia	Sbf1	SRX6864129	this study (locality: Norin stream, 43.053475 N, 17.595780 E)
NER.2	Neretva	Croatia	Sbf1	SRX6864130	this study (locality: Norin stream, 43.053475 N, 17.595780 E)
NEV.1	Nevezis	Lithuania	Pst1	SRX3997981	1
NEV.2	Nevezis	Lithuania	Pst1	SRX3997990	1
NID.1	Nideraach	Switzerland	Sbf1	SRS257619	9 (locality described in ref. 4)
NID.2	Nideraach	Switzerland	Sbf1	SRS257621	9 (locality described in ref. 4)
OBR.1	Oberriet	Switzerland	Sbf1	SRS1271421	8
OBR.2	Oberriet	Switzerland	Sbf1	SRS1271424	8
ROM.1	Romanshorn	Switzerland	Sbf1	SRS257608	9 (locality described in ref. 4)
ROM.2	Romanshorn	Switzerland	Sbf1	SRS257609	9 (locality described in ref. 4)
SAS.1	Saint-Sulpice	Switzerland	Sbf1	SRX1038830	3 (locality described in ref. 4)
SAS.2	Saint-Sulpice	Switzerland	Sbf1	SRX1038827	3 (locality described in ref. 4)
SKA.1	Skadar	Albania	Pst1	SRX3997967	1
SKA.2	Skadar	Albania	Pst1	SRX3997966	1
SLI.1	Nevan	Russia	Pst1	SRX3998001	1
SLI.2	Nevan	Russia	Pst1	SRX3997998	1
SLU.1	Sluch	Ukraine	Pst1	SRX3997982	1
SLU.2	Sluch	Ukraine	Pst1	SRX3998063	1
SOR.1	Sorgue	France	Sbf1	SRX6084942	2
SOR.2	Sorgue	France	Sbf1	SRX6084941	2
STP.1	Saint-Pourcain-sur-Sioule	France	Sbf1	SRX6101711	2
STP.2	Saint-Pourcain-sur-Sioule	France	Sbf1	SRX6101716	2
SZO.1	Szödliget	Hungary	Pst1	SRX3997987	1
SZO.2	Szödliget	Hungary	Pst1	SRX3997986	1
TET.1	Teterev	Ukraine	Pst1	SRX3998065	1
TET.2	Teterev	Ukraine	Pst1	SRX3998062	1
VAL.1	Valence	France	Pst1	SRX3997984	1
VAL.2	Valence	France	Pst1	SRX3997985	1
VAT.1	Vättern	Sweden	Pst1	SRX3998050	1
VAT.2	Vättern	Sweden	Pst1	SRX3998051	1

Supplementary Fig. 1.

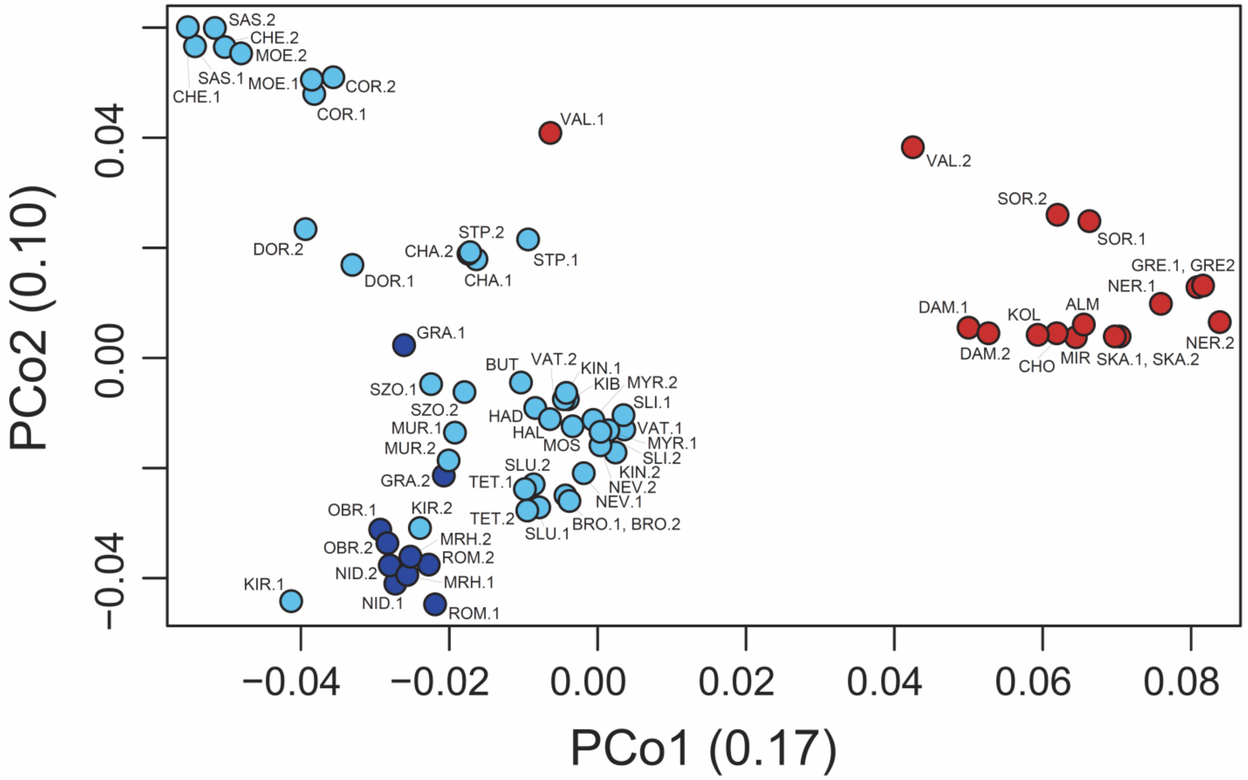

Supplementary Fig. 1. Genetic relationship among the European stickleback individuals included in the phylogeny (Fig. 1), as expressed by Principal Coordinates Analysis (PcoA) of individual SNP genotype data. The individuals are mapped along the first two ordination axes (Principal Coordinates 1 and 2). The relative importance of these axes (eigenvalues) is given in parentheses. The color coding follows Fig. 1. Further methodological detail is given in the Methods.

Supplementary Fig. 2.

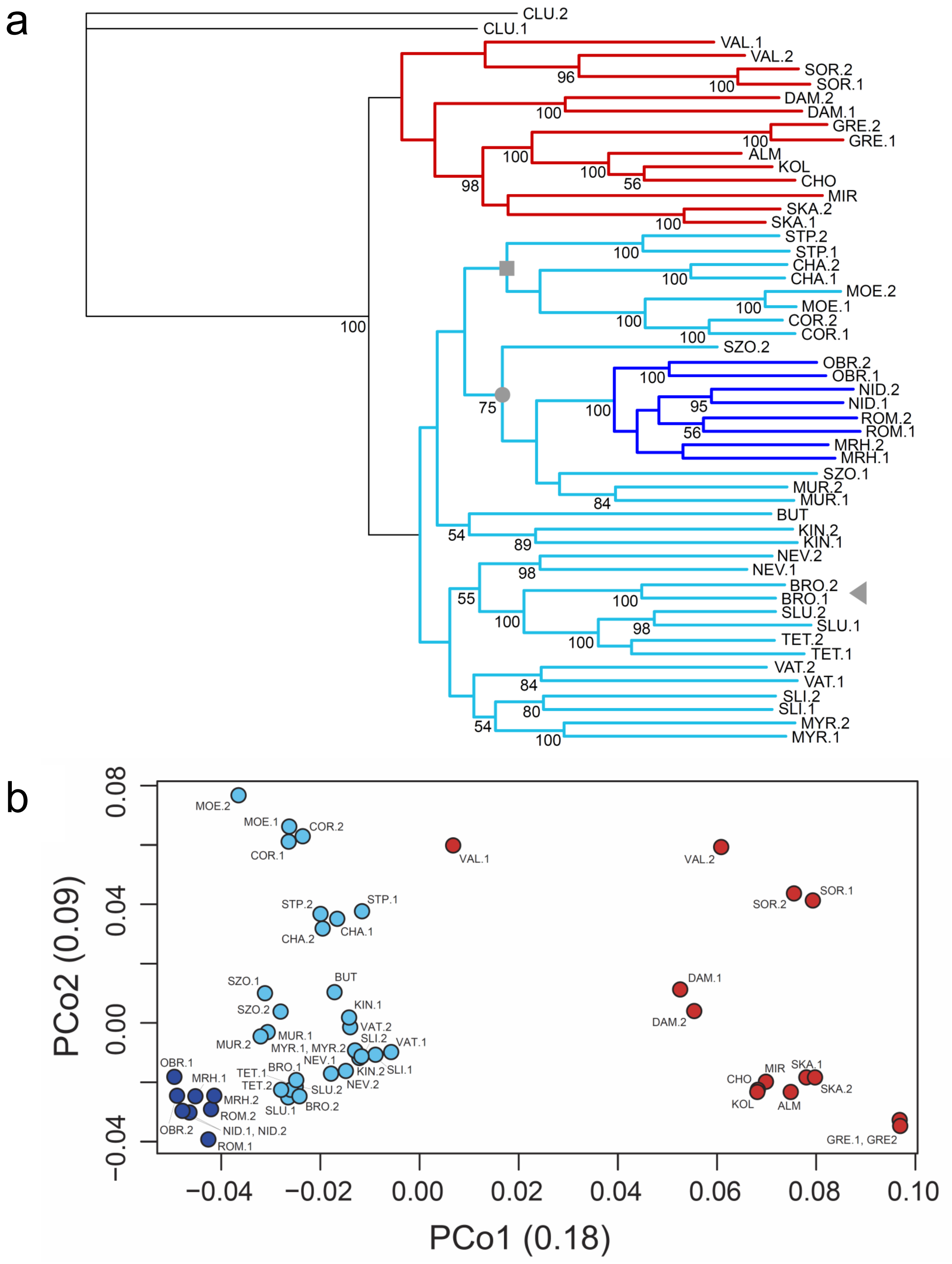

Supplementary Fig. 2. Phylogeny (a) and genetic ordination (b) of European threespine stickleback populations. The graphics correspond to Fig. 1 and Supplementary Fig. 1, except that the underlying analyses were performed by considering only data sets available to the investigation of Marques et al.[2].

Supplementary References

1. Fang, B., Merilä, J., Ribeiro, F., Alexandre, C. M. & Momigliano, P. Worldwide phylogeny of three-spined sticklebacks. *Mol. Phylogenet. Evol.* **127**, 613–625 (2018).
2. Marques, D. A., Lucek, K., Sousa, V. C., Excoffier, L. & Seehausen, O. Admixture between old lineages facilitated contemporary ecological speciation in Lake Constance stickleback. *Nat. Commun.* **10**, 4240 (2019).
3. Roesti, M., Kueng, B., Moser, D. & Berner, D. The genomics of ecological vicariance in threespine stickleback fish. *Nat. Commun.* **6**, 8767 (2015).
4. Berner, D., Roesti, M., Hendry, A. P. & Salzburger, W. Constraints on speciation suggested by comparing lake-stream stickleback divergence across two continents. *Mol. Ecol*. **19**, 4963–4978 (2010).
5. Roesti, M., Gavrilets, S., Hendry, A. P., Salzburger, W. & Berner, D. The genomic signature of parallel adaptation from shared genetic variation. *Mol. Ecol.* **23**, 3944-3956 (2014).
6. Moser, D., Roesti, M. & Berner, D. Repeated lake-stream divergence in stickleback life history within a Central European lake basin. *PLoS One* **7**, e50620 (2012).
7. Ferchaud, A.-L. & Hansen, M. M. The impact of selection, gene flow and demographic history on heterogeneous genomic divergence: threespine sticklebacks in divergent environments. *Mol. Ecol.* **25**, 238-259 (2016).
8. Marques, D. A., Lucek, K., Meier, J. I., Mwaiko, S., Wagner, C. E., Excoffier, L. & Seehausen, O. Genomics of rapid incipient speciation in sympatric threespine stickleback. *PLoS Genet.* **12**, e1005887 (2016).
9. Roesti, M., Salzburger, W. & Berner, D. Uninformative polymorphisms bias genome scans for signatures of selection. *BMC Evol. Biol.* **12,** 94 (2012).
