# Supplementary material for: Re-evaluating the evidence for facilitation of stickleback speciation by admixture in the Lake Constance basin
Source: Nat Commun. 2021 May 14;12:2806. doi: 10.1038/s41467-021-23092-1 (PMC8121923; doi:10.1038/s41467-021-23092-1)
Supplement: Supplementary file 2 — Description of Additional Supplementary Files [file 41467_2021_23092_MOESM2_ESM.pdf]

Description of Additional Supplementary Files

File Name: Supplementary Code 1
Description: Compilation of all code used for sequence file manipulation, genotyping, SNP detection, and phylogenetic and ordination analyses.
