# Supplementary material for: Re-evaluating the evidence for facilitation of stickleback speciation by admixture in the Lake Constance basin
Source: Nat Commun. 2021 May 14;12:2806. doi: 10.1038/s41467-021-23092-1 (PMC8121923; doi:10.1038/s41467-021-23092-1)
Supplement: Supplementary file 4 — Reporting Summary [file 41467_2021_23092_MOESM4_ESM.pdf]

nature research

Corresponding author(s): Daniel Berner

Last updated by author(s): Mar 31, 2021

Reporting Summary

Nature Research wishes to improve the reproducibility of the work that we publish. This form provides structure for consistency and transparency in reporting. For further information on Nature Research policies, see our Editorial Policies and the Editorial Policy Checklist.

Statistics

For all statistical analyses, confirm that the following items are present in the figure legend, table legend, main text, or Methods section.

n/a	Confirmed	
☒	☐	The exact sample size (*n*) for each experimental group/condition, given as a discrete number and unit of measurement
☒	☐	A statement on whether measurements were taken from distinct samples or whether the same sample was measured repeatedly
☒	☐	The statistical test(s) used AND whether they are one- or two-sided<br>*Only common tests should be described solely by name; describe more complex techniques in the Methods section.*
☒	☐	A description of all covariates tested
☒	☐	A description of any assumptions or corrections, such as tests of normality and adjustment for multiple comparisons
☒	☐	A full description of the statistical parameters including central tendency (e.g. means) or other basic estimates (e.g. regression coefficient) AND variation (e.g. standard deviation) or associated estimates of uncertainty (e.g. confidence intervals)
☒	☐	For null hypothesis testing, the test statistic (e.g. *F*, *t*, *r*) with confidence intervals, effect sizes, degrees of freedom and *P* value noted<br>*Give P values as exact values whenever suitable.*
☒	☐	For Bayesian analysis, information on the choice of priors and Markov chain Monte Carlo settings
☒	☐	For hierarchical and complex designs, identification of the appropriate level for tests and full reporting of outcomes
☒	☐	Estimates of effect sizes (e.g. Cohen's *d*, Pearson's *r*), indicating how they were calculated

Our web collection on statistics for biologists contains articles on many of the points above.

Software and code

Policy information about availability of computer code

Data collection	Single-nucleotide polymorphism (SNP) data underlying the genetic analyses were generated by processing fastq short read data with functions from the ShortRead (v 1.24.0), Rsamtools (v 1.18.3), and stringr (v 1.4.0) packages for R (v 3.1.3 and 3.6.1). All R code was written by the author and is available as Supplementary file. Short read alignment was performed with Novoalign (v 3.00) using parameter settings also described in the Supplementary file.
Data analysis	Phylogenetic and ordination analysis based on the SNP data were performed with the ape (v 5.0) and phangorn (2.5.5) packages for R (v 3.6.1). All R code was written by the author and is available as Supplementary file.

For manuscripts utilizing custom algorithms or software that are central to the research but not yet described in published literature, software must be made available to editors and reviewers. We strongly encourage code deposition in a community repository (e.g. GitHub). See the Nature Research guidelines for submitting code & software for further information.

Data

Policy information about availability of data

All manuscripts must include a data availability statement. This statement should provide the following information, where applicable:

- Accession codes, unique identifiers, or web links for publicly available datasets
- A list of figures that have associated raw data
- A description of any restrictions on data availability

All raw Illumina sequence data used for genetic analysis are available from the NCBI Sequence Read Archive under the accession numbers listed in Supplementary Table 1.

Field-specific reporting

Please select the one below that is the best fit for your research. If you are not sure, read the appropriate sections before making your selection.

- [ ] Life sciences
- [ ] Behavioural & social sciences
- [x] Ecological, evolutionary & environmental sciences

For a reference copy of the document with all sections, see nature.com/documents/nr-reporting-summary-flat.pdf

Ecological, evolutionary & environmental sciences study design

All studies must disclose on these points even when the disclosure is negative.

Study description	Analysis of genetic relationships among threespine stickleback (Gasterosteus aculeatus) fish from Europe, based on raw short read data from this and previous studies. Involves data from 69 individuals from 39 geographic locations.
Research sample	Involves new short read data from four geographic locations (two individuals each). The rest of the data used was published previously and is publicly available: Roesti et al. 2012 BMC Evol. Biol.; Roesti et al. 2015 Nat. Commun.; Ferchaud & Hansen 2016 Mol. Ecol.; Fang et al. 2018 Mol. Phylogenet. Evol.; Marques et al. 2019 Nat. Commun.
Sampling strategy	A sample size of two individuals per location was used when possible. This sample size is common in phylogenetics. Moreover, for numerous locations considered, no more than two individuals were available (all locations from Fang et al. 2018).
Data collection	This study involved no data collection. Data were either derived from source material from existing, published collections (Berner et al. 2010 Mol. Ecol.; Moser et al. 2012 PLoS One), or were obtained from the NCBI sequence read archive (accession codes given in Supplementary Table 1).
Timing and spatial scale	This study involved no data collection.
Data exclusions	No data were excluded.
Reproducibility	SNP calling was performed in two modes, the second one particularly defensive (higher haplotype coverage and inter-SNP spacing requirements). Both approaches led to similar results supporting the same conclusions. Moreover, the study reports a supplementary analysis performed by excluding a subset of individuals not available to the authors of the study targeted by my Matters Arising. This also produced qualitatively similar findings as with the full data set.
Randomization	Where possible, I selected individual short read data sets based on maximum file size, thus maximizing individual read depth. Since file size is determined purely by technical aspects of library preperation and sequencing and is unrelated to any biological feature of an individual, the choice of study individuals represents random sampling.
Blinding	The study did not involve any measurement or attribution of qualities to individuals, hence blinding was not relevant and was not performed.

Did the study involve field work? [ ] Yes [x] No

Reporting for specific materials, systems and methods

We require information from authors about some types of materials, experimental systems and methods used in many studies. Here, indicate whether each material, system or method listed is relevant to your study. If you are not sure if a list item applies to your research, read the appropriate section before selecting a response.

Materials & experimental systems

n/a	Involved in the study	
[x]	[ ]	Antibodies
[x]	[ ]	Eukaryotic cell lines
[x]	[ ]	Palaeontology and archaeology
[ ]	[x]	Animals and other organisms
[x]	[ ]	Human research participants
[x]	[ ]	Clinical data
[x]	[ ]	Dual use research of concern

Methods

n/a	Involved in the study	
[x]	[ ]	ChIP-seq
[x]	[ ]	Flow cytometry
[x]	[ ]	MRI-based neuroimaging

Animals and other organisms

Policy information about studies involving animals; ARRIVE guidelines recommended for reporting animal research

Laboratory animals	The study did not involve laboratory animals.

Wild animals	The study did not involve wild animals. All data were derived from preserved tissue from previously published collections.
Field-collected samples	The study did not involve samples collected from the field.
Ethics oversight	This study required no ethical approvement or guidance because it used publicly available sequence read data, or such data were generated based on tissue avaialble from previous, already published collections.

Note that full information on the approval of the study protocol must also be provided in the manuscript.
